# Hypodermic Medication

**Published:** 1871-06

**Authors:** W. A. Greene

**Affiliations:** Americus, Ga.


### HYPODERMIC MEDICATION.

BY DR. W. A. GREENE, M. D., OF AMERICUS, GA.

(*Continued from last No.*)

I will now leave the discussion of this interesting part of the subject, to speak briefly of the physiological and therapeutical effects of calabar bean employed hypodermically, which presents many interesting and curious developments, and opens an extensive field for research and experimental investigation.

During the last few years, M. Bournville, house surgeon to the Paris Hospitals, has employed it with care and studied its effects on several diseases. Among the results may be mentioned the following: Every one knows that the calabar bean, when instilled into the eye, produces a very remarkable contraction of the pupils. Now he states that it is surprising to observe that, when it is injected under the skin, there ensues, on the contrary, a dilatation of the pupils, or else the occular diaphragm remains unchanged. A second point which he notices, is the antagonism between the calabar bean and atropine. He verified this phenomenon in six guinea pigs. After having injected the bean, atropine was injected. The animals did not succumb when both medicaments were injected in fit proportions; but two or three days afterwards, a dose of the bean equal to that which had been at first employed being injected into the surviving animals, *they all died.* This obviously shows the antagonistic action of atropine against the calabar bean. He also confirmed these experiments on the human subject, and treated with more or less success epilepsy, chorea and tetanus. Dr. Eben Watson states that in all his experiments, the pupils remained either unchanged or were dilated. Mr. T. Wharton Jones, in a suggestive paper, published in the Practitioner, for September, 1869, refers to this surprising fact and says, "the calabar bean is the direct antagonist of atropia, and asserts that "the local application of the drug causes contraction of the veins and atropia of the arteries." Dr. Alois Monti, of the H. Annis child's hospital, reports three cases out of five of trismus neonatorium cured by this remedy. He prefers the hypodermic method, because he considers the internal use uncertain. He repeats these injections every ten or fifteen minutes, until the spasms cease; and then intermits them even for several hours until the cramp returns again. For new born children he uses one-tenth grain of the extract for a dose, and goes up to one-third, one-half or a whole grain a day. Older children can commence with one-third of a grain for a dose. For internal use, one to four grains may be given. In the Richmond and Louisville Journal for August, 1870, Dr. F. C. Wilson reports a case of epilepsy in which there had been six successive convulsions at intervals of twenty minutes, relieved by ten minims of the tincture of calabar bean used subcutaneously.

In the Medical Examiner for July, 1870, Dr. Newman reports a case of tetanus successfully treated by the hypodermic injection of calabar bean.

The Glasgow Medical Journal (1869 p. 300) publishes two cases treated by Dr. Wm. Fenwick. The first case died in less than thirty-six hours after the commencement of treatment, owing to the culpable negligence of the attendants; the directions being very ineffectually carried out. The second case recovered, and is especially interesting, owing to the suspension of the medicine being three times followed by increase of symptoms, which yielded to its renewed exhibition.

Dr. Eben Watson, in the Practitioner of September 1870, in a paper "on treatment of tetanus by the calabar bean," details several cases—eighteen, I believe, in which he reports ten recoveries—and always found the remedy to act decidedly better when introduced under the skin; in fact considers this the most essential point in its administration. Thus we perceive a wide and interesting field for the hypodermic use of this medicine. My own opportunities have been limited for its exhibition. In a case of epilepsy, I found it exceedingly efficacious. I have drawn from reliable and trustworthy authority, and hope sufficient evidence has been adduced to induce my professional brethren to test its virtue and efficacy.

I will now proceed to speak of veratrum veride, a most familiar remedy—and am sure there is no medicine in our Materia Medica that deserves more praise, and whose effects on the human system are more uniform and consequently more to be relied upon. All are acquainted with its effects on the human system when administered by the mouth, and it is therefoae to its hypodermic use that I invite your attention. It effectually takes the place of the lancet when thus administered, and acts more rapidly, more energetically and more satisfactorily. When rapid arterial excitement or tumultuous action of the heart is sought to be controlled, this medicine injected under the skin in proper doses accomplishes a desideratum most devoutly to be wished for. In the convulsions of children, the result of high arterial excitement, so trying to the physician, and frightful to the relatives and friends, we possess a certain remedy in veratrum. I begin the injection under the skin at the back of the neck, in half drop doses, which may be increased to two drops, combined with a few drops of warm water—and if its constitutional effects are not produced in ten or twenty minutes, cautiously repeat the dose. I frequently combine with the veratrum five or ten drops of Squibbs' compound liquor of opium. In the first stage of pneumonia, where it is important to produce rapid sedation, or where the stomach fails to tolerâte it, I find it invaluable. I use it cautiously, beginning with one or two drops. I will here relate a case in point, occurring a few days since in the practice of my friend Dr. George F. Cooper, of this city, in his own words :

"Lucy, mulatto girl aged sixteen, attacked with double pneumonia—saw her fourth day—pulse 120—respiration labored and rapid. Injected one drop of Norwood's veratrum under the skin of arm. In twenty minutes pulse reduced to 118. Injected two drops more. In twenty minutes pulse reduced to 90—then ordered it continued by mouth every three hours, in four or six drop doses. Her recovery, though not rapid, was uninterrupted and complete in eight days. No blister or other medication used, except two or three purgative doses of mercury."

In puerperal convulsions veratrum is a most valuable remedy. I will give two recent cases in which I employed it, thereby better conveying to the mind the mode of its administration and its effects on tha system. January 8th, 1871, called to see Mrs. W., aged 38, in consultation with my friend Dr. Bird, two miles from town. I arrived at her bedside 11 o'clock, p. m. She lacked two months to the completion of her term. Nothing unusual had occurred during gestation, her general health being uninterrupted—had borne several children, and never required the presence of a physician during her labors. She had been having convulsions since early in the morning, at intervals of twenty to forty minutes; had taken large doses of laudanum and been kept under chloroform all day—bowels been freely opened with enemeta, pulse very large and as hard a one as I ever felt; it beat 130 times a minute. The os uteri was just large enough to admit the point of my index finger, and very rigid. Had a dreadful convulsion a few moments after my arrival. I immediately opened a large median vein in her right arm, and allowed blood to flow until her pulse had sunk very low. She had no return of the convulsions for about an hour, remaining nearly comfortable—when her circulation began to increase, restlessness came on, and she had a most violent eclampsia, followed by apoplectic stertor, from which she partially recovered in half an hour. I immediately gave her ten drops of the solution of morphine and atropine, which had the effect to quiet her at once—and she rested and slept comfortably for one hour and forty minutes. She awoke much excited and another convulsion came on. I determined then to give her veratrum subcutaneously. By this time she refused to open her mouth or swallow, the stupor being more profound than before. This was fifteen minutes past two o'clock—pulse 140—injected two drops of Norwood's veratrum. In ten minutes pulse reduced to 130, with no disposition to go lower—2½ o'clock injected three drops more. In fifteen minutes slight impression noted on pulse—convulsions strongly threatened. Fifteen minutes of 3 o'clock injected four drops. 3 a. m. pulse reduced to 90 beats, breathing much improved, and sleeping quietly. Awoke at 6 a. m. perfectly rational, with ability to swallow. Directed following prescription:

℞—Bromide potassium . . ℥ i.
Water, . . . . ℥ viij.

Sig. One table-spoonful every six or eight hours in half glass of water.

The circulation was kept under control for two or three days by occasional doses of veratrum, combined with the bromide. She continued to do well until February 12th, when I was sent for and she was delivered of a dead fœtus of eight mouths. Labor was rapid and easy, from which she recovered as usual. Now could anything be more charming than the prompt result of this medicine, and that after the much-vaunted remedy—bleeding, seemed about to fail? The veratrum did not produce the slightest nausea. Dr. Bird rendered valuable assistance in conducting this case.

Since this article was commenced, I have witnessed a most terrible case of puerperal convulsions, the treatment of which presents many interesting features. I will merely give my notes of the case as taken down at the bedside:

Called March 5th, at 11 a. m., to see Mrs. H. in consultation with her attending physician, Dr. Hudson. She was a woman of a very susceptible, nervous constitution, and labored under constitutional irritation from the commencement of her pregnancy. She was 25 years old, and this her first labor—had been married about twelve months. Was expecting her labor every hour, the time being out—had complained of intense headache for several days. About 4 a. m., she awoke her husband to do something for her head. By the time he could make a light, she had a convulsion—had no uterine pain whatever. The convulsions returned every ten or fifteen minutes until Dr. H. arrived (about an hour afterwards), and gave a large dose of morphine and put her under chloroform—when the convulsions were delayed an hour or more in their intervals. About 10½ o'clock they became more rapid, and I was sent for. She had a convulsion when I arrived, and immediately sunk into a stertorous, apoplectic sleep. I felt that the fated blow had been struck, effusion or extravasation having taken place. There was no uterine action—the os uteri was firmly contracted, very rigid, and the perineum excessively hard and thick—the vertex in the first position. The fœtal heart could be distinctly heard. Withdrew the chloroform, bled her 18 ounces, and gave ten drops of solution morphine and atropia fifteen minutes thereafter—had five convulsions in quick succession, the last one lightest. Eleven and half a. m., pulse 145—injected one drop veratrum in a few drops of water—in ten minutes pulse reduced to 130—in twenty minutes 120—12¾, convulsion—eight minutes to 1 p. m., gave one drop veratrum, combined with eight drops solution of morphine and atropia—pulse 115—1-25 p. m., pulse 135 and convulsion came on—bled her 12 ounces—1-32 injected one half grain ergotine, combined with two drops of veratrum in thigh, pulse being 135. In eighteen minutes, pulse reduced to 120—twenty minutes no change—twenty minutes more pulse 118—1½, convulsion—3-7 pulse 128, os uteri yet contracted and no uterine pain—coma being profound and stertorous, rapid breathing—child alive—3 p. m., ruptured members and injected half grain of ergotine in thigh, combined with three drops of veratrum. 3-45 pulse 120, injected three drops of veratrum; in ten minutes, pulse 116, and gave half grain ergotine. Some uterine contractions—waters all passed off—os dilated size half dollar—5 p. m., severe convulsion—5-40 half grain ergotine injected in abdomen—uterine contractions decidedly increased—coma *very* profound—6-45 p. m. convulsion—uterine contraction going on—7 p. m. head of fœtus descended so that forceps could be applied, which was immediately done, and a 12 pound child extracted. Mother died in one hour, and child doing well.

This woman never spoke or exhibited the smallest sign of reason or sensation from the moment of invasion, but sank at once into the stertorous, apoplectic sleep that finally led to the sleep of death. Notwithstanding these functions of the brain were oppressed from the beginning—the hemispheres, the cerebellum and the tubercular quadrigemina remaining extinct as to power—yet our remedies did much good. This is very interesting. The veratrum controlled the circulation and lengthened the intervals of the convulsions from ten or fifteen minutes to one hour and twenty minutes. The ergotine speedily and promptly excited uterine contractions and thus enabled us to save the child. We could not have done so without it—the mother's case already a hopeless one—the child alive as the strokes of its heart indicated, profound coma and total inability to swallow, and no effort of the womb whatever to expel its contents—it did seem that the mother would succumb with a live child in her womb. The ergotine injected under the skin of the thigh and abdomen aroused this dormant uterus, and it responded by sending the fœtal head down into the vagina, where it could be reached by forceps and delivered. I am indebted to Dr. Hudson for valuable assistance in this case, and courtesy in obtaining its history.

I have found the addition of veratrum with the morphine and atropine solution speedily relieve distressing paroxysms of asthma that had resisted all other treatment. I have never seen any reference to the hypodermic use of veratrum, and hope by this imperfect account of its employment, to attract the attention of the profession, that its value and efficacy may be further developed and illustrated.

I come now to speak of ergotine. In controlling excessive menorrhagia, there is no remedy equal to it, used hypodermically. Severe cases of this character that had resisted various treatments and strongly threatened death, have yielded almost instantly to the hypodermic injection of ergotine dissolved in equal parts of water and glycerine. In my hands it has produced remarkable effect in the hemorrhage following delivery. Under these circumstances of sudden and alarming bleeding, we want a remedy that speedily affects the system. I present it to the profession and ask for it a faithful trial. The dose is from one-third, one half or even one grain dissolved in equal parts of glycerine and water, and the injections repeated every fifteen or twenty minutes. The hypodermic use of ergotine in aneurisms is attracting considerable attention. I have had no opportunity of testing its value in these cases, b[illegible] [illegible]ill mention two cases reported by Langenback.

The first was that of a man aged 45, with a pulsating tumor in the neck, the size of a fist, the pulsations in which greatly annoyed the patient. On the 6th of January, 1869, Prof. L. made a first subcutaneous injection of 0.03 grm. (half a grain) watery extract of ergot on a level with the aneurism. After this the pulsations became weaker and the tumor diminished in size. Between the 6th January and the 17th February, there were injected at regular intervals of three days two grammes of ergotine in doses varying from 0.03 to 0.18 grammes (half a grain to three grains.) The tumor was notably diminished in size and pulsations were less strong.

The second case was that of a man aged 42, who had for twenty years a pulsating tumor of the radial artery of the size of a hazel nut three centimetres from the hand. Prof. L. injected 0.15 grammes of ergotine between the skin and the tumor; the next day the aneurism could not be detected. It reappeared momentarily in consequence of a slight effort made by the patient, but it had completely disappeared at the end of eight days. The editors of the Archives Generales (Nov. 1869,) from which journal this account was condensed (see Am. Med. Journal 1870,) justly remarks that "these results seem extraordinary and to have been obtained very promptly." I hope to hear of further experiment with this remedy in this disease. The hypodermic injection of the salts of mercury in the treatment of constitutional syphilis is engaging the attention of the profession, especially in the East. I have had no patients recently who would submit to its use, and therefore have no personal experience with it. I am, however, well satisfied of its utility and efficacy, and shall not hesitate to employ it. I will briefly glance at what eminent men say of it by way of attracting the attention of the profession to its employment, for a few experiments conducted by master hands in all that relates to therapeutics aud pharmacy are more important and reliable than a far greater number would be detailed by persons of less authority.

M. Diday at a meeting of the Lyons' Medical Society, detailed the result of twelve cases of syphillis treated by hypodermic injection of corrosive sublimate. His solution consisted of distilled water 45 grammes, corrosive sublimate, glycerine, of each ten centigrammes. Two or three injections were practical daily, the mean quantity of the sublimate introduced being seven or eight millegrammes. The back is used for the operation because pain is less complained of there—or if the patient injects himself, the sides of the chest and anterior or external parts of the thighs are used, alternating these regions so that the puncturos do not succeed each other too rapidly, or approach each other too closely.

He says: "I must admit great embarrassment in pronouncing an opinion on this new method. In fact, there is no occasion to pronounce any positive opinion on a remedy which has so recently made its appearance. At first, one is struck and seduced by the promptitude of some of the cures which it effects." M. Diday believes it most advantageous in the squamous form of syphilis and warns against its use in the ulcerative forms. In none of his cases was the slightest mercurial effect of the mouth produced. Dr. T. J. Walker, British Medical Journal, records the results of his experience, in ten cases in which it was fairly tried, and found immediate improvement after one or two injections. He says: "In the first case, after three weeks of treatment and the injection of only 7-10 of a grain of bichlorid of mercury, the change in the condition of the patient was surprising.

In case 2d, these symptoms which were rather of the secondary type were all relieved after six injections, and the tertiary symptoms yielded with unusual rapidity to the iodide of potassa when it was commenced. The solution he employed amounted to $\frac{1}{8}$ gr. of corrosive sublimate dissolved in ten drops of water and glycerine. This solution may be used every day. Dr. Walker states that the pain and inflammation at the seat of puncture are comparatively slight, and considers the results he has attained justify its trial in large institutions.

The hypodermic syringe is an exceedingly useful little instrument for drawing out liquors for diagnostic and other purposes. It has been made use of for obtaining blood for examination from cholera patients. It has been used for the operation of transfusion, and, with slight modification, preferred above all other instruments. Dr. Mader of Vienna used it in making a diagnosis in a case of ascites, in which a doubt existed whether chronic peritonitis or cirrhosis hepatis caused the dropsy. The character of the liquid proved it to be a case of cirrhosis. He also pumped out a chronic serous exudation from the pericardial sac of an aged female: the first operation yielded a few ounces of liquid and relieved the urgent symptoms; at a second trial, three ounces were removed. Mader thinks the operation deserves much regard, as it is easily performed, and attended with comparatively little danger. In these operations the canula should be provided with a stop-cock, so that air may not enter when the body of the syringe is detached in order to empty it, and the body of the syringe should be made of greater capacity.

I will not lengthen this already too extended review by referring to other remedies that are used hypodermically, and mentioned in the beginning of this article. The field is wide open, and the opportunity for research and investigation inviting, and offers a rich harvest to the industrious lover of science. I feel that I could not practice medicine without the hypodermic syringe—know that I am indebted to it for many a night's sleep and relief from restless anxiety. Instead of worrying all night, as I have many times done, with cases of colic, delirium tremens, gout, rheumatism, convulsions, neuralgia, nausea and nervous excitability, I have relieved my patients in fifteen or twenty minutes and returned to my bed.

